# LAL Regulators *SCO0877* and *SCO7173* as Pleiotropic Modulators of Phosphate Starvation Response and Actinorhodin Biosynthesis in *Streptomyces coelicolor*

**DOI:** 10.1371/journal.pone.0031475

**Published:** 2012-02-20

**Authors:** Susana M. Guerra, Antonio Rodríguez-García, Javier Santos-Aberturas, Cláudia M. Vicente, Tamara D. Payero, Juan F. Martín, Jesús F. Aparicio

**Affiliations:** 1 Institute of Biotechnology INBIOTEC, León, Spain; 2 Area of Microbiology, University of León, León, Spain; National Institutes of Health, United States of America

## Abstract

LAL regulators (Large ATP-binding regulators of the LuxR family) constitute a poorly studied family of transcriptional regulators. Several regulators of this class have been identified in antibiotic and other secondary metabolite gene clusters from actinomycetes, thus they have been considered pathway-specific regulators. In this study we have obtained two disruption mutants of LAL genes from *S. coelicolor* (Δ0877 and Δ7173). Both mutants were deficient in the production of the polyketide antibiotic actinorhodin, and antibiotic production was restored upon gene complementation of the mutants. The use of whole-genome DNA microarrays and quantitative PCRs enabled the analysis of the transcriptome of both mutants in comparison with the wild type. Our results indicate that the LAL regulators under study act globally affecting various cellular processes, and amongst them the phosphate starvation response and the biosynthesis of the blue-pigmented antibiotic actinorhodin. Both regulators act as negative modulators of the expression of the two-component *phoRP* system and as positive regulators of actinorhodin biosynthesis. To our knowledge this is the first characterization of LAL regulators with wide implications in *Streptomyces* metabolism.

## Introduction

Streptomycetes are filamentous soil bacteria that have a complex life cycle that involves differentiation and sporulation. These bacteria have attracted great interest due to their well-known ability to produce a great variety of secondary metabolites including therapeutic molecules like antibiotics, immunosuppressants, or anticancer agents. Production of these compounds is regulated in response to nutritional status alteration and a variety of environmental conditions, and hence occurs in a growth-phase-dependent manner, at the transition between the rapid growth phase and the stationary growth phase, and usually accompanied by morphological differentiation [1].

The control of secondary metabolite production is a complex process involving multiple levels of regulation. While the lowest level is composed by regulatory genes that only affect a single antibiotic biosynthetic pathway, the highest level includes genes that exert a pleiotropic control over both development and secondary metabolism [2]. Pathway-specific regulatory genes are usually found within their respective antibiotic biosynthesis gene cluster, a feature that has greatly facilitated their study, but higher regulators can be located far away in the chromosome, thus making it difficult to infer their targets.

*Streptomyces coelicolor* is the model organism among streptomycetes. Its 8.7 Mb linear genome contains more than 20 secondary metabolite gene clusters [3], including those for the blue pigmented polyketide actinorhodin (Act), the red-pigmented tripyrrole undecylprodigosin (Red), and the lipopeptide calcium dependent antibiotic (CDA) [4]. It also contains a significant number of genes which seem to encode regulators that need to be assigned a function. Particularly, LAL (Large ATP-binding regulators of the LuxR family)-encoding genes, which have been poorly studied. This family of regulators, typified by the regulator of the maltose regulon in *Escherichia coli*, MalT [5], is characterized by the unusual size of its members, and the presence of an N-terminal ATP/GTP-binding domain easily identified by the presence of the conserved Walker A motif [6], and a C-terminal LuxR-like DNA-binding domain characterized by a conserved helix-turn-helix motif. Several regulators of the LAL family have been identified in antibiotic and other secondary metabolite gene clusters from actinomycetes, including PikD from the pikromycin pathway in *S. venezuelae* [7], RapH from the rapamycin pathway in *S. hygroscopicus* [8], NysRI and NysRIII from the nystatin pathway in *S. noursei* [9], and AmphRI and AmphRIII from the amphotericin pathway in *S. nodosus* [10], [11], among others. The former are pathway-specific regulators, although it is conceivable that LAL regulators could play a role in higher steps of the regulatory cascade.

The availability of the genome sequence, together with the development of efficient methods for genome-wide analysis of expression profiles using DNA microarrays [12], can enable the analysis of the global effect that pleiotropic regulatory gene mutants have, and thus identify the genes affected by each mutation which will help us to establish regulatory networks.

In this study we have constructed *S. coelicolor* mutants in two LAL regulatory genes, *SCO0877* and *SCO7173*, and used DNA microarrays to perform genome-wide transcriptome profiling of both mutants in order to understand their pleiotropic implications. Comparative transcriptome analysis of wild type and these mutants enabled us to gain insights into the aspects controlled by these poorly studied regulators. We show here that these LAL regulators affect phosphate starvation response and actinorhodin biosynthesis, among other processes.

## Results and Discussion

### Experimental design

A complete analysis of *S. coelicolor* genome allows the identification of 23 Open Reading Frames (ORFs) that could be assigned to the LuxR family of transcriptional regulators. Among them, 14 seem to belong to the LAL family of regulators [13] given that they share the common features of large size, HTH motif, and ATP binding site (Table 1).

**Table 1:** Regulators of the LuxR family identified in the *S. coelicolor* genome.

Gene/protein	Number ofaminoacids	ATP/GTP bindingmotif	Putative LALregulator
SCO0132	919	YES	YES
SCO0712	941		
SCO0877	888	YES	YES
SCO1331	780	YES	YES
SCO1351	912	YES	YES
SCO2686	338		
SCO3665	327		
SCO3666	394		
SCO4263	1251		
SCO4276	223		
SCO4768	203		
SCO5065	943	YES	YES
SCO5506	1091	YES	YES
SCO6029	220		
SCO6193	943	YES	YES
SCO6334	892	YES	YES
SCO6993	606	YES	
SCO7093	932	YES	YES
SCO7134	923	YES	YES
SCO7137	924	YES	YES
SCO7143	937	YES	YES
SCO7173	908	YES	YES
SCO7295	988	YES	YES

Since production of secondary metabolites is known to be enhanced by factors occurring in complex media [14], our working hypothesis was that differential expression of a given regulatory gene in complex and defined media could be evidence of its implication in secondary metabolism. We expected to find good expression of LAL positive regulators in complex medium given that secondary metabolite production is normally triggered in such media. On the contrary, such expression would be negatively affected in defined medium (minimal medium), where the production of secondary metabolites is generally quite poor.

**Figure 1:**
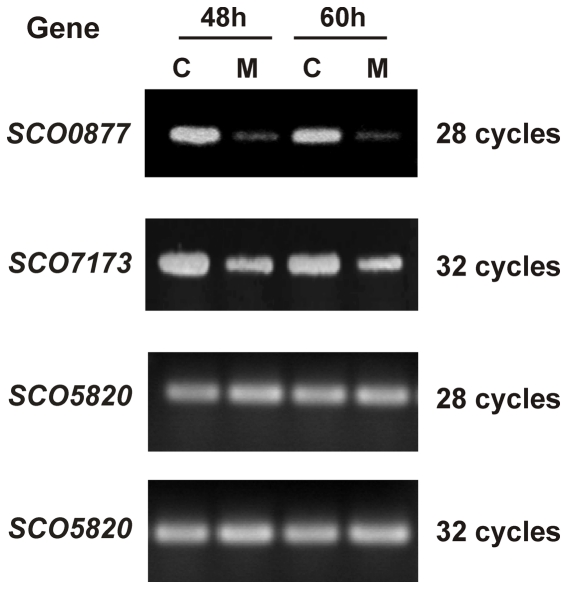
Gene expression analysis of the genes *SCO0877* and *SCO7173* by RT-PCR. Analysis was carried out on *S.coelicolor* M145 strain as indicated in the Methods section after 28 (*SCO0877*) or 32 (*SCO7173*) PCR amplification cycles. RNA was extracted from cultures after growth for 48 h and 60 h in defined (M) and complex (C) MG medium. The identity of each amplified product was corroborated by direct sequencing. The absence of contaminating DNA in the RNA samples was assessed by PCR. Transcription of the *hrdB* gene (*SCO5820*) was assessed as an internal control (bottom).

Total RNA was prepared from *S. coelicolor* wild type strain after growth for 48 h and 60 h in defined and complex MG medium and used as template for gene expression analysis by reverse transcriptase-polymerase chain reaction (RT-PCR). Primers for RT-PCR were specific to sequences within the LAL genes (Table S1) and were designed to produce cDNAs of approximately 400–600 bp. A primer pair designed to amplify a cDNA of the *hrdB* gene was used as an internal control. Transcripts were analyzed after a number of PCR cycles that ranged from 26 to 37 (depending on the gene). These analyses were carried out at least in three independent biological replicates for each primer pair. Following this strategy, we identified two LAL genes, *SCO0877* and *SCO7173*, which constituted good candidates for study. They showed a clear higher expression in complex medium than in defined medium at both time points (Fig. 1).

### Construction of LAL mutants

In order to determine the function of *SCO0877* or *SCO7173*, we inactivated them independently by using the REDIRECT gene replacement technology as indicated in Materials and Methods. Double-crossover mutants were screened by apramycin resistance and kanamycin sensitivity. These (about 10%) were verified by both PCR and Southern blot analysis (not shown).

The new mutant strains *S. coelicolor* Δ0877 and *S. coelicolor* Δ7173 had growth and morphological characteristics identical to those of *S. coelicolor* M145 when grown on TBO, MS or YEPD solid media, or MG liquid medium. The spore counts of the three strains were similar after growth for 9 days at 30°C on TBO plates. All strains grew well, showing an identical growth curve.

### Deletion mutants show reduced actinorhodin production

**Figure 2:**
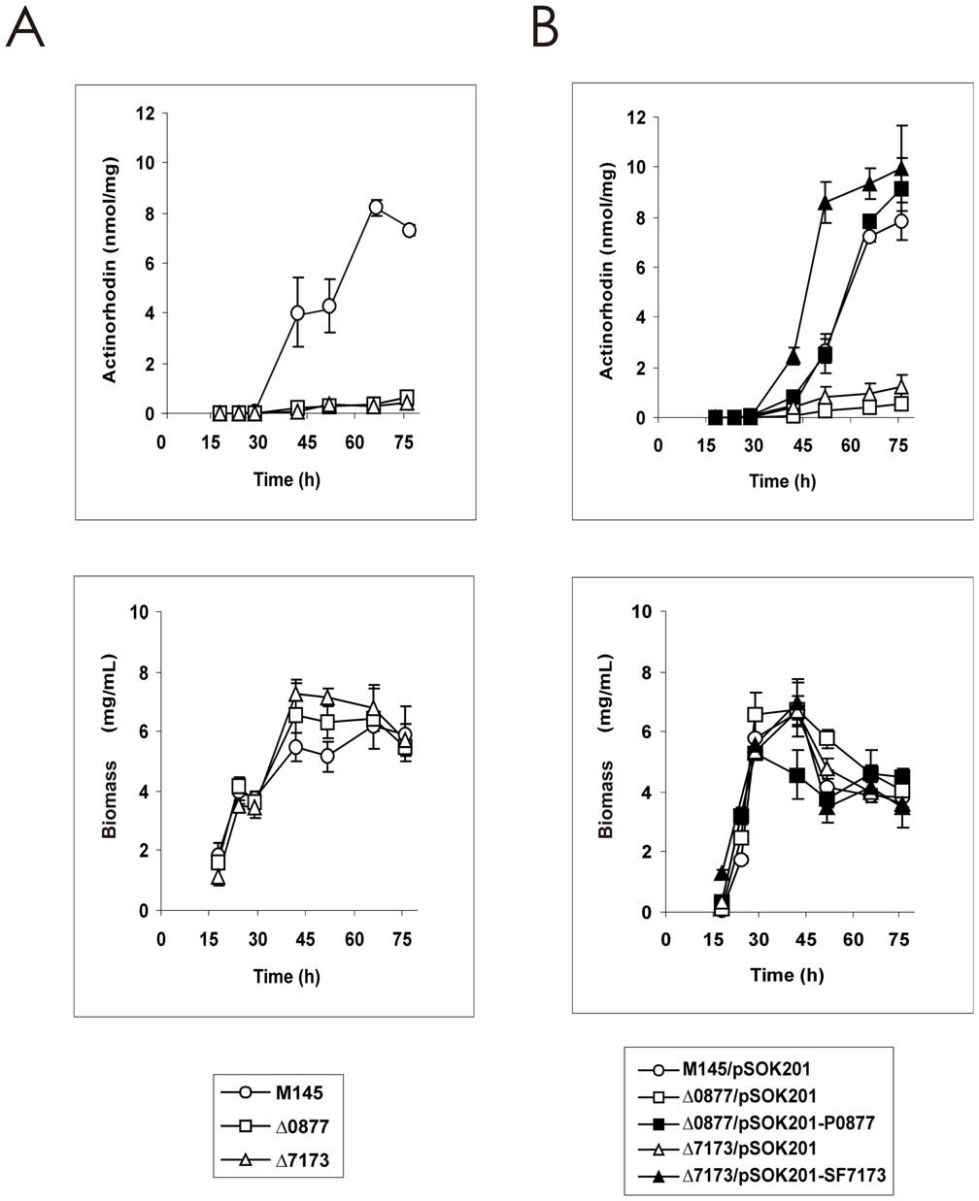
Actinorhodin production kinetics of strains M145, Δ0877 and Δ7173, and gene complementation. Specific production of actinorhodin (expressed as nmol of antibiotic per mg of dry weight), in complex MG medium. A) Open circles indicate production by the wild type strain, open squares the production observed in the Δ0877 strain, and open triangles the production observed in the strain Δ7173. B) Antibiotic production upon gene complementation. For control strains (i.e. transformed with pSOK201), symbols are as in (A) panels. Solid squares indicate production by the Δ0877 strain complemented with *SCO0877*, and solid triangles the production observed in the strain Δ7173 complemented with *SCO7173*. Data are the average of three flasks. Vertical bars indicate the standard deviation values. Growth curves are shown at the bottom panels.

Growth pattern and antibiotic titers of the two mutant and the parental strains were measured during a three-day liquid culture in complex MG medium. Disruption of the gene did not significantly alter growth kinetics (Fig. 2). Interestingly, while production of the lipopeptide calcium dependent antibiotic (CDA), and the red-pigmented tripyrrole undecylprodigiosin remained similar in the three strains (not shown), the production of the blue pigmented polyketide actinorhodin was severely reduced in both mutants (Fig. 2).

*SCO0877* is the first gene of an apparent three gene operon, and *SCO7173* overlaps the 3′-end of a gene encoding a putative 89-aminoacid hypothetical protein, so their inactivation could have had an impact on flanking genes, and the phenotype observed could be attributed to a polar effect. In order to discard such possibility, gene complementation was performed. In both cases, genetic complementation with only the LAL-encoding gene was sufficient to restore actinorhodin synthesis (Fig. 2, see Materials and Methods), thus suggesting that each mutant phenotype was due to the LAL-encoding gene knockout.

Taken together, these results strongly suggest a role for both LAL regulators in modulation of antibiotic synthesis in *S. coelicolor*, particularly of actinorhodin. In order to investigate this aspect, we decided to use DNA microarrays for the assessment of the genes whose transcription was differentially affected in the mutants. This microarray approach involved the use of cDNA prepared from total RNA isolated from complex MG medium, and genomic DNA (gDNA) as a universal reference for all hybridizations [15], [16].

### Identification of genes with an altered expression profile in LAL mutants

Following the statistical criteria described in Materials and Methods, 322 genes showed differential transcription in at least one of the mutants, and were selected for further analysis (Appendix S1). 121 of them were differentially transcribed in the *S. coelicolor* Δ0877 mutant while 263 showed differences in mutant *S. coelicolor* Δ7173. Interestingly, all the genes in common (62) followed the same pattern in the differential expression profile, regarding up- or down-regulation, in both mutants. Selected genes differentially transcribed are included in Tables S2 and S3.

#### Genes involved in amino acid metabolism, transcription and translation

This group includes 23 genes that showed differential transcription in at least one of the mutants. These genes code for enzymes involved in amino acid metabolism (12 genes), ribosomal proteins (4 genes), proteins involved in transcription (6 genes, including 5 sigma factors), and a putative lysyl-tRNA synthetase (*SCO3397*) (Tables S2 and S3). Interestingly, the four genes that code for ribosomal proteins (*rplA*, *rpsD*, *rpsL* and *rpmD*) and five genes involved in transcription (sigma factors *sigU*, *sigT*, *sigQ*, *SCO4769*, and the transcription accessory protein *SCO6743*), showed decreased transcription levels in both mutants, although this result is statistically significant (SS; see Materials and Methods) only in mutant *S. coelicolor* Δ7173 (Table S3). On the other hand, sigma factor *sigJ* showed increased transcript levels in both mutants, being SS only in mutant *S. coelicolor* Δ0877 (Table S2). Only three SS genes are common to both mutants, namely aminotransferase *SCO1054*, methionine synthase *SCO1657*, and the lysyl-tRNA synthetase (*SCO3397*), and all of them showed increased transcript levels in the mutants (Table S2), which indicates a down-regulation of these genes mediated by both LAL regulators.

#### Genes involved in nucleotide and coenzyme metabolism, and DNA replication, recombination, and repair

Sixteen genes falling into this category were found to be differentially transcribed in the mutants. Eight of them are involved in DNA replication, recombination and repair. Of these, helicases *SCO5166* and *SCO5439*, nucleases *SCO3347* and *uvrA*, DNA gyrase (*gyrA*), and the recombination regulator *recX* showed reduced transcription in the mutants (all SS in mutant *S. coelicolor* Δ7173) (Table S3), while helicase *SCO1167* and DNA ligase *SCO1202* displayed the opposite behavior, both being SS in *S. coelicolor* Δ0877 mutant (Table S2). Six genes are involved in coenzyme metabolism, and the remaining two in purine metabolism. Of all these SS genes, only two were common to both mutants, helicase *SCO1167* which showed increased transcription, and *recX* regulator which showed decreased transcription.

#### Respiration and energy production genes

Nine genes belonging to this group were found to be differentially transcribed in the mutants. These include three genes involved in oxidative phosphorylation (*atpE*, *atpG*, and *cyoE*) which showed decreased transcription in both mutants, the first two SS in mutant *S. coelicolor* Δ7173, and *cyoE* in *S. coelicolor* Δ0877 mutant (Table S3). The remaining six genes affected are involved in energy production, and show increased transcription in the mutants. These, include two glycerophosphoryl diester phosphodiesterases (*SCO1419* and *SCO1968*), one of the nitrate reductases (*narH3*), an acyl-CoA synthetase (*SCO5842*), and acetate kinase (*ackA*). Although all these genes share the same transcription pattern in both mutants, only one (*SCO1968*) is SS in both (Table S2).

#### Genes involved in cell envelope biosynthesis and morphological differentiation

This group includes 17 genes that showed differential transcription in at least one of the mutants. These genes, code for enzymes involved in cell envelope biosynthesis (9 genes), morphological differentiation (7 genes), and also an FtsW-like protein putatively involved in cell division during sporulation (*SCO3846*) (Tables S2 and S3). Among them, those related to morphological differentiation are particularly interesting because in *Streptomyces* morphological differentiation is usually accompanied by physiological differentiation [17]. Our results indicate that the extracytoplasmic sigma factor *sigU* that causes a delay in aerial mycelium formation when overexpressed [18] is down-regulated in strain Δ7173, while the regulator *nsdA* that indirectly represses actinorhodin biosynthesis [19] is up-regulated in strain Δ0877. *ramS*, which encodes a polypeptide that is used as starter material for the lantibiotic-like peptide SapB [20], is up-regulated in both mutants, whereas the essential gene for sporulation *whiA* [21] is up-regulated in strain Δ0877.

The differential expression of genes involved in morphological differentiation was somehow unexpected given that both mutants were morphologically identical to the wild type, and that traditionally it has been considered that *S. coelicolor* was not able to differentiate in liquid cultures. However, recent quantitative proteomic studies have demonstrated that *S. coelicolor* non-sporulating liquid cultures undergo a rather complex differentiation process [22].

#### Carbohydrate metabolism genes

While no gene belonging to this category is differentially transcribed in mutant Δ0877, seven genes show a SS differential transcription in *S. coelicolor* Δ7173 mutant (Tables S2 and S3). These include several sugar hydrolases, a transketolase A1, and one of the paralogs of phosphoglycerate mutase (*pgm1*). Proteomic studies have shown that this enzyme is activated by PhoP under phosphate limitation, and this mechanism has been proposed as a cellular compensation to substrate limitation [16]. Our results indicate that *pgm1* is down-regulated by LAL 7173, which could indicate a connection to phosphate starvation response in this bacterium (see below).

#### Lipid metabolism genes

Eight genes related to lipid metabolism are differentially transcribed in at least one of the mutants. These include a putative 3-ketoacyl-ACP/CoA reductase (*SCO0330*), a lipoic acid synthetase (*SCO2194*), and an enoyl CoA hydratase (*SCO4384*), which are involved in fatty acid biosynthesis, or genes related to steroid biosynthesis like 2C-methyl-D-erythriol 2,4-cyclodiphosphate synthase (*SCO4234*), among others. Interestingly, all these genes showed increased transcription in both mutants (Table S2).

#### Phosphate starvation response genes

Twenty six genes differentially expressed in the mutants were conspicuously related to phosphate metabolism or to phosphate starvation response [16], [23], all of them showing increased transcription (Table S2). They include the *phoR*-*phoP* two-component system and the phosphate transduction signal modulator *phoU*, glycerophosphoryl diester phosphodiesterase homologs (*SCO1419* and *SCO1968*), or the phosphate specific transporters *pstSA*. Other genes affected include *neuAB*, which are involved in putative phosphorous-free teichuronic acid biosynthesis as a substitute for phosphate-rich teichoic acids, genes encoding twin-arginine translocation (Tat) dependent exported proteins *SCO1196*, *SCO1633*, *SCO6691* and *SCO7631* [24], and the phosphate scavenging secreted phytase *SCO7697*. Most of these genes are members of the PHO regulon [16], that is they are directly controlled by the two-component PhoP-PhoR system, which drives the cellular response to phosphate scarcity [25]. DNA binding of phosphorylated PhoP response regulator to its operators (PHO boxes) occurs following phosphate depletion in the culture media and this binding controls the expression of phosphate regulated genes [16], [26]. Thus, the differential transcription of all these genes in both mutants suggests that both regulators control the phosphate starvation response, maybe through the PhoP-PhoR system itself, which is autoregulated [26], [27].

Interestingly, among the genes affected by the mutation of LAL 0877 is the methallotionein-encoding *mtpA* gene whose expression has been described to be up-regulated when Pi is limiting but independently of PhoP [16], [28]. In the mutant *S. coelicolor* Δ0877 the expression of this gene is triggered, which is evidence of down-regulation, whereas LAL 7173 does not seem to control this gene.

Recent studies have revealed that all these genes are also controlled by *afsS* [23], and also that there is a direct control of *afsS* by PhoP [29]. Strikingly, although not SS, this gene showed decreased transcription in both LAL mutants which is evidence for positive regulation. This result is especially clear in mutant *S. coelicolor* Δ7173, which showed a *p-*value of 0.0083.

**Figure 3:**
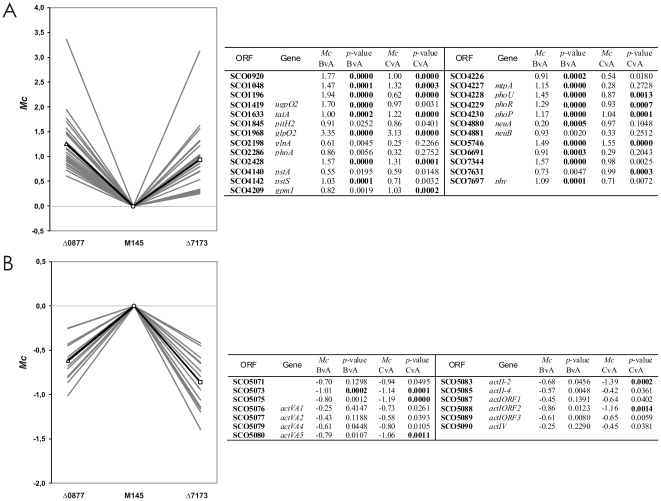
Transcription profiles of phosphate starvation response and *act* genes. Differential transcription values *Mc* were obtained by subtracting mutant *Mg* values from M145 *Mg* values. A) 25 phosphate starvation response genes are included in the plot. These are: SCOs *0920*, *1048*, *1196*, *1419*, *1633*, *1845*, *1968*, *2198*, *2286*, *2428*, *4140*, *4142*, *4209*, *4226*–*4230*, *4880*–*4881*, *5746*, *6691*, *7344*, *7631*, and *7697*. Gray lines are the plots of transcription values for individual genes in a given mutant, the black lines are the mean profiles for the mutant strains. *Mc* and *p-*values for the contrasts between the indicated conditions (A: *S. coelicolor* A3(2) M145. B: *S. coelicolor* Δ0877. and C: *S. coelicolor* Δ7173 strains) are shown at the right. B) Differential transcription values for 13 genes of the *act* cluster. These include: SCOs *5071*, *5073*, *5075*–*5077*, *5079*–*5080*, *5083*, *5085*, *5087*–*5090*.

Overall, our results indicate that phosphate starvation response genes are down-regulated by the LAL regulators under study. Figure 3A shows the transcription profile of 25 phosphate starvation response genes, including 15 whose transcription has been demonstrated to be directly controlled by PhoP (see Table S2). This profile indicates that the phosphate starvation response system is tightly controlled by both regulators LAL 0877 and LAL 7173. Thus, both produce the opposite effect on the expression of these genes than PhoP.

#### Actinorhodin biosynthetic genes

In MG medium, *S. coelicolor* M145 begins synthesis of prodigiosins at the end of the exponential phase, and actinorhodin production is delayed some 15 hours (Fig. 2). Microarray analysis revealed that while genes belonging to the prodigiosins *red* cluster were not significantly affected by the mutations, the enoyl reductase *actVI-orf2* showed SS reduced transcription in both mutants. This gene has been shown by gene disruption studies to be involved in actinorhodin production as its disruption prevented actinorhodin biosynthesis [30]. Four additional genes belonging to the actinorhodin biosynthetic cluster showed SS decreased transcription in the *S. coelicolor* Δ7173 mutant (Table S2). Moreover, analysis of all the genes of the *act* cluster, and application of a limma uncorrected *p-*value<0.05, revealed that 13 genes of the cluster, including the SARP (Streptomyces Antibiotic Regulatory Protein) activator *actII*-*orf4*, showed decreased transcription in both mutants (Fig. 3B). This result explains the phenotypes of the mutants regarding actinorhodin production, and indicates up-regulation of *act* genes by both regulators, which is especially significant in LAL 7173.

Earlier studies in *S. lividans* and *S. natalensis* using deletion mutants of the *phoRP* system [25], [27] in liquid complex media revealed that disruption of this regulatory locus lead to overproduction of actinorhodin or pimaricin, respectively. Given that both regulators down-regulate the *phoRP* system, it is possible that the observed phenotypic effect upon gene mutation could be produced via the PhoR-PhoP system. Moreover, although not SS, both mutants show decreased transcription of the gene *afsS*, this gene codes for a small “sigma-like” protein that regulates antibiotic biosynthesis. Thus, overexpression of *afsS* in *S. coelicolor* and *S. lividans* caused actinorhodin overproduction [31], [32], while disruption of this gene blocked production completely [23]. Since LAL mutants show reduced expression of the *afsS* gene, this could also contribute to the reduced actinorhodin production observed upon mutation.

Additionally to *act* genes, the epoxide hydrolase *cpkE* of the cryptic polyketide synthase cluster *cpk* [33] was also found to be up-regulated by LAL 7173.

#### Regulatory genes

As described here, a large set of genes with diverse functions are under the control of regulators LAL 0877 and LAL 7173, including several regulatory genes included in the categories listed above. This prompted us to analyze other possible transcriptional regulators differentially expressed in the mutants, as these could be mediators of the regulatory control. A complete list of regulatory genes whose expression is affected in the mutants is presented in Tables S2 and S3.

A total of 37 transcriptional regulators show a significant differential transcription in the mutants when compared with the parental strain, 19 of them in *S. coelicolor* Δ0877, and 27 in *S. coelicolor* Δ7173. Such a large number reflects the pleiotropic nature of both regulators, and probably justifies all the biological processes affected by the mutations (see functional categories listed above).

Among the regulators controlled by LAL 0877, is interesting to highlight NsdA, which is a negative regulator of differentiation and antibiotic synthesis in *S. coelicolor* [19]. Since, *S. coelicolor* Δ0877 mutants show increased expression of the *nsdA* gene, this could contribute to the reduced actinorhodin production observed upon mutation. In addition, both the sigma factor *sigU* and its cognate anti-sigma factor *rsuA* showed reduced transcription in both mutants. Given that *rsuA* deletion strains are significantly delayed in actinorhodin biosynthesis [18], this could also contribute to the reduced actinorhodin production observed.

### Validation of microarray results by using quantitative RT-PCR

Quantitative RT-PCR was used on reversed transcribed RNA samples to confirm that differential expression indicated by the microarray data was supported by an independent method. A total of 11 genes showing high Mc and a *p*-value of less than 0.0002 were chosen for analysis. The selected genes covered a wide range of expression, including up-regulation and down-regulation. These included five genes of the *pho* regulon (*glpQ*-SCO1968, *phoU*, *phoR*, *phoP*, *SCO1196*), four related to actinorhodin biosynthesis (*actVI-orf2*, *actVI-orf4*, *actII*-2, *SCO5746*), and two unrelated to the former processes, *SCO1209* that encodes a putative acyl-CoA dehydrogenase, and *SCO7318*, which encodes a putative membrane protein. Overall, the qRT-PCR data and microarray data showed a good concordance (Figure S1). The range of dynamics for relative log_2_ 2^−ΔΔCt^ obtained from qRT-PCRs (−3 to +5.3) was significantly higher than Mc values from microarrays (−1.4 to +3.3), indicating that qRT-PCRs are more sensitive. This probably reflects on the Pearson's correlation coefficient for the plot (*r* ^2^ = 0.823), resulting in a lower value than what could be expected.

### Control of LAL regulators on *phoP* expression

**Figure 4:**
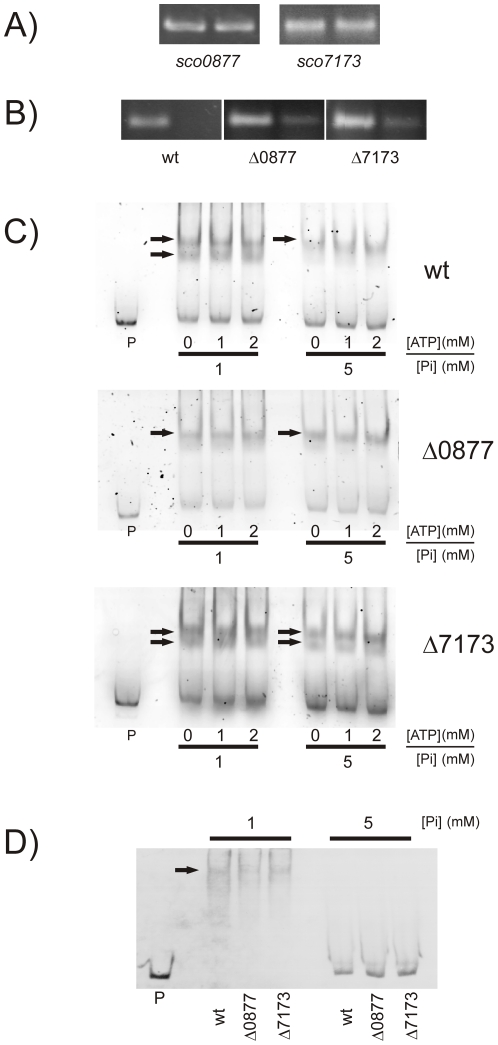
Control of LAL regulators on *phoP* expression. A) Gene expression analysis of the LAL genes by RT-PCR. Analysis was carried out on *S.coelicolor* M145 and the mutant strains as indicated in the Methods section after 28 (*SCO0877*) and 32 (*SCO7173*) PCR amplification cycles. RNA was extracted from cultures after growth for 48 h under conditions of phosphate abundance (5 mM) (right lanes) or limitation (1 mM) (left lanes). The identity of each amplified product was corroborated by direct sequencing. The absence of contaminating DNA in the RNA samples was assessed by PCR. B) Gene expression analysis of *phoP* by RT-PCR. Analysis was carried out after 30 PCR amplification cycles. Conditions for RNA isolation were as in A. C) Electrophoretic mobility analysis (EMSA) of protein extract binding to the *phoP* promoter region. The arrows indicate the DNA–protein complexes. All experiments were carried out with 2 ng labeled DNA probe. The probe (P) was incubated for 15 min at 30°C with 30 µg of protein extract prepared from cultures of *S. coelicolor* M145, *S. coelicolor* Δ0877 and *S. coelicolor* Δ7173 grown for 48 h on MG medium containing 1 or 5 mM phosphate, in the presence of 0, 1, or 2 mM ATP. D) EMSA of protein binding to the *phoRP* promoter region. Conditions for binding were as in C. No differences were observed when incubation was carried out in the presence of 1, or 2 mM ATP.

One of the most interesting outcomes of our transcriptomic studies was that both LAL regulators act as negative regulators of the *phoRP* system expression. Expression of this system is driven by a promoter situated upstream the *phoR* gene, leading to a bicistronic transcript, but *phoP* can also be transcribed alone from a promoter located at the 3′-end of *phoR* [28]. Expression from that promoter has been described to be under the control of a transcriptional repressor in *S. lividans* in conditions of phosphate abundance [34]. So it was interesting to determine whether the level of expression of the LAL regulators studied varied with phosphate availability. Total RNA was prepared from *S. coelicolor* wild type strain after growth for 48 h under conditions of phosphate proficiency (5 mM) or limitation (1 mM) and used as template for gene expression analysis by RT-PCR. Results showed no variation in the level of expression of the LAL regulators (Fig. 4A). Additionally, and given that the expression of *phoP* is strongly repressed in phosphate proficiency [16], the same type of experiment was performed to determine whether the level of expression of *phoP* in the mutant strains still varied with phosphate availability. Results showed that while *phoP* expression was completely repressed in the wild type strain under conditions of phosphate abundance, some expression was still detected in the mutants (Fig. 4B), thus suggesting that the phosphate control observed in the wild type is deregulated in the mutants. Interestingly, expression was higher in the mutant strains than in the wild type under conditions of phosphate limitation, thus corroborating our transcriptomic results (Figure 4B).

In order to test whether these regulatory proteins were able to interact directly with the *phoP* promoter region, band-shifting experiments were set up using crude protein extracts prepared from the wild-type strain and the mutants grown under conditions of phosphate abundance or limitation for 48 hours. These studies were carried out in the presence and absence of ATP. The results are shown in Figure 4C. Under conditions of phosphate limitation (1 mM) two shifted bands were observed in both the wild type and mutant Δ7173 strains, whereas in mutant Δ0877 the lower band disappeared. This suggests a direct interaction between LAL 0877, or another regulator positively modulated by LAL 0877, and the *phoP* promoter. No major differences in binding were observed in the presence or absence of 1 mM or 2 mM ATP. Under conditions of phosphate abundance (5 mM), we observed just one shifted band in both the wild type and mutant Δ0877 strains, whereas in mutant Δ7173 an extra shifted band was detected. This could be produced by a protein component that is normally repressed by LAL 7173 and is made evident in the mutant.

When we carried out the same type of experiment with the *phoRP* promoter region (Fig. 4D) a single band shifting was observed in all strains. Again, no major differences on binding were observed in the presence or absence of 1 mM or 2 mM ATP (not shown). Conversely, there was no retardation band at phosphate abundance. These results indicate that the LAL regulators studied are not able to bind the *phoRP* promoter region under the conditions assayed, thus suggesting that their negative effect on *phoRP* expression could be indirect. Furthermore, the shifted band observed must be produced by a component of the crude extracts that is more abundant under conditions of phosphate limitation, characteristic that correlates well with the regulator PhoP. Thus it is feasible that the observed retardation band is produced by PhoP. PhoP is able to bind *phoRP* promoter [26], and its expression is repressed under conditions of phosphate abundance [16]. Further experimental analyses will be required to test these possibilities.

To ensure that the observed shifts were specific, competition experiments with the same unlabelled probes were performed. In both cases, the addition of cold probe reduced the intensity of shifted bands, whereas the addition of a non-specific competitor (see Materials and Methods) did not produce any change in their intensity (not shown). Interestingly, these band-shifting experiments with the two promoters that drive the expression of *phoP*, demonstrate that there are various DNA-binding proteins that participate in the regulation of *phoP* transcription, and that the control over the two promoters is rather different, thus reflecting the intricate regulatory network centered on this regulator.

### Conclusion

One of the main characteristics of the soil-dweller *Streptomyces* is its ability to produce a vast array of secondary metabolites. The production of these compounds is generally dependent on growth phase and involves the expression of clustered regulatory and biosynthetic genes, but besides these genes other genes, largely situated outside of biosynthetic gene clusters, also affect the biosynthesis of such compounds. Secondary metabolism is, therefore, a complex playground where global and pathway-specific regulators form a web of interactions that finally results in metabolite production. Many global regulators exert pleiotropic control over both development and secondary metabolism (e.g. *abs*, *afs* and *bld* genes), and this turned out to be the case of the LAL regulators studied. This pleiotropic behavior is reflected in the large number of regulatory genes affected by the mutations.

Our results indicate that the LAL regulators under study act globally affecting various cellular processes, and amongst them the phosphate starvation response in *S. coelicolor*. Both produce the opposite effect on the expression of the PHO regulon genes than PhoP, and this effect is probably mediated by the *phoRP* system given that both down-regulate the system itself. Interestingly, cross regulation between phosphate deprivation and nitrogen metabolism has been described, and demonstrated to be mediated by PhoP [16]. However, and despite being the PhoRP system repressed by both LAL regulators, there are no nitrogen genes affected in the LAL mutants. This probably indicates that other regulators participate in the regulation of nitrogen metabolism besides the *phoRP* system.

It is noteworthy that both regulators affect independently the two component system *phoRP*. Band shifting experiments with the two promoters that drive the expression of *phoP* have shown that there are various DNA-binding proteins that participate in the regulation of *phoP* transcription. The fact that both LAL regulators control genes of the PHO regulon in the same direction (i.e. down-regulation), may well reflect that the phosphate starvation response is too important for *Streptomyces* as to leave it in hands of a single path, and requires a fine and balanced tuning. This is especially significant bearing in mind the changing nature of the nutritional conditions in its natural habitat, the soil.

Another cellular process affected by both regulators is the biosynthesis of the blue-pigmented antibiotic actinorhodin. In both mutants, the production of actinorhodin is severely impaired. Transcriptome analysis of the mutants revealed that both regulators control transcription of *actVI-orf2* (*SCO5073*), while LAL 7173 controls also other four genes of the *act* cluster. In all cases transcription is reduced in the mutants, which could account for the phenotype observed. Although at this stage a direct interaction of any of the LAL regulators to *act* promoters cannot be excluded, it is likely that the effect could be produced via other regulators such as PhoP, AfsS, NsdA or RsuA. Several other results of our transcriptomic studies could explain the reduction of actinorhodin biosynthesis in the mutant strains: i) both mutants show increased transcription of the *phoRP* system that is known to be a negative regulator of actinorhodin biosynthesis [25]; ii) both mutants show reduced expression of the *afsS* gene, which is a positive regulator of actinorhodin production; iii) *nsdA* transcription, which encodes a negative regulator of differentiation and antibiotic synthesis in *S. coelicolor* [19], is increased in the mutants, especially in strain Δ0877; iv) *rsuA* transcription, which is an anti-sigma factor whose expression has been correlated with the correct onset of actinorhodin production [18], is reduced in both mutants, especially in strain Δ7173; v) both mutants show increased transcription of genes involved in fatty acid biosynthesis such as 3-ketoacyl-ACP/CoA reductase (*SCO0330*), lipoic acid synthetase (*SCO2194*), or enoyl CoA hydratase (*SCO4384*) which could compete for precursors required for actinorhodin biosynthesis. Further work is required to test these possibilities. In any case, we have shown that both LAL regulators are master modulators of both phosphate starvation response and actinorhodin biosynthesis in *S. coelicolor*. The findings reported here should provide important clues to understand the intertwined regulatory machinery that modulates antibiotic biosynthesis in *Streptomyces*.

## Materials and Methods

### Bacterial strains, cloning vectors and cultivation

*S. coelicolor* A3(2) strains M145, Δ0877 and Δ7173 were cultured on TBO medium [35] to obtain high concentration spore suspensions. Alternatively they were grown on solid MS [36] or YEPD (yeast extract 10 g/l, peptone 20 g/l, glucose 20 g/l) media. *Escherichia coli* DH5α was used as general cloning host. *E. coli* ET12567 [pUZ8002] was used as donor in intergeneric conjugations. *E. coli* BW25113 was used as the host for Red recombination [37] and to propagate plasmid pIJ790 [38]. The replicative vector pSOK201 [39] was used for gene complementation. Cultures were performed at 30°C and 300 rpm in defined MG medium [40] containing 140 mM glucose, 1 mM phosphate, and 7.6 mM (NH_4_)SO_4_. Cultures containing an additional 5 g/l yeast extract but no added phosphate were used for the complex medium condition. Analysis of the phosphate content of the later medium resulted in 0.98 mM which is equivalent to the defined medium. 50 ml of the corresponding MG medium in 0.5 L baffled flasks were inoculated at a density of 4×10^6^ spores/ml for dispersed growth. Antibiotic assays were carried out as described elsewhere [36].

### Genetic procedures

Standard genetic techniques with *E. coli* and *in vitro* DNA manipulations were as described by Sambrook and Russell [41]. Recombinant DNA techniques in *Streptomyces* species and isolation of *Streptomyces* total DNA were performed as previously described [36]. Southern hybridization was carried out with probes labeled with digoxigenin by using the DIG DNA labeling kit (Roche Biochemicals). Intergeneric conjugation between *E. coli* ET12567 [pUZ8002] and *S. coelicolor* strains was performed as described [42].

### Construction of mutants Δ0877 and Δ7173

Deletion of *SCO0877* from *S. coelicolor* was made by replacing the wild-type gene with a cassette containing an apramycin selective marker using a PCR based system [38]. The plasmid pIJ773 containing the apramycin resistance gene (*aac(3)IV*) and the *oriT* replication origin was used as a template. The mutant was constructed using the oligonucleotides 5′-ccggacgccggccgtccccctttagagtgggcttctgtgATTCCGGGGATCCGTCGACC-3′ and 5′-*gaactcttcactccagatggttacgtttcgcatgcgtca*TGTAGGCTGGAGCTGCTTC-3′ as the forward and reverse primers respectively (the sequence identical to the DNA segment upstream from the start codon of *SCO0877* is underlined and in lower case and the sequence identical to the segment downstream from the stop codon of *SCO0877* is in lower case italics). These two long PCR primers (59 nt and 58 nt) were designed to produce a deletion of *SCO0877* just after its start codon leaving only its stop codon behind. The 3′ sequence of each primer matches the right or left end of the disruption cassette (the sequence is shown uppercase in both primers). The extended resistance cassette was amplified by PCR and *E. coli* BW25113/pIJ790 bearing cosmid StM1 was electro-transformed with this cassette. The isolated mutant cosmid was introduced into non-methylating *E. coli* ET12567 containing the RP4 derivative pUZ8002. The mutant cosmid was then transferred to *S. coelicolor* M145 by intergeneric conjugation. Double cross-over exconjugants were screened for their kanamycin sensitivity and apramycin resistance.

Mutant Δ7173 was constructed following the same strategy, using the oligonucleotides 5′-gggcgcttcgtccggaccggcatcggtgcccacggcatgATTCCGGGGATCCGTCGACC-3′ and 5′-*aggtggcgcagctgatgggcgctacggaggaggagttca*TGTAGGCTGGAGCTGCTTC-3′ as the forward and reverse primers respectively, and the cosmid template St9A4.

In both cases, mutants were verified by both PCR and Southern blot analysis.

### Gene complementation

Mutants were complemented by reintroduction of the wild-type alleles as follows. In the case of *SCO0877*, a 3323 bp *Eco*RI DNA fragment containing the entire *SCO0877* gene including its own promoter was ligated into an *Eco*RI-cut pSOK201 [39], to yield pSOK201-P0877. This plasmid was then transferred by conjugation from *E. coli* ET12567 [pUZ8002] to the *S. coelicolor* Δ0877 mutant. pSOK201 was also introduced into the same strain, and into the parental strain *S. coelicolor* M145 as controls. In the case of *SCO7173*, a 2912 bp PCR amplified DNA fragment comprising 120 bp upstream of the start codon (to include an *Eco*RV site) and the entire *SCO7173* gene was cloned into pGEM®-T Easy. The primers used were 5′-GGCCGATATCAGAGGTGTGC-3′ and 5′-GAGGGAATTCGTGGACCAGGTGCGTGCC-3′. The resulting plasmid was cut with *Spe*I and *Eco*RV (the *Spe*I site belongs to pGEM), and ligated to a 91 bp PCR amplified DNA segment containing SF14 promoter from pAR9331 [43]. The primers used were 5′-CCTCACTAGTATTAATGAGTTACGTAGACCTACGC-3′ and 5′-CCTGGATATCCTAATCGAGTATTGATTGTAGCTCAC-3′.Then, a 2988 bp *Eco*RI DNA fragment containing *SCO7173* under the control of SF14 promoter was ligated into an *Eco*RI-cut pSOK201 [39], to yield pSOK201-SF7173. This plasmid was then transferred into *S. coelicolor* strains as indicated above. Reintroduction of wild-type genes was, in both cases, confirmed by PCR.

### Nucleic acid extractions

RNA was extracted as described [44] after 48 h of growth. Briefly, eight ml from liquid cultures were used mixed with one volume 40% (v/v) glycerol, and mycelia were harvested by centrifugation and immediately frozen by immersion in liquid nitrogen. Frozen mycelium was then broken by shearing in a mortar, and the frozen lysate was added to buffer RLT (Qiagen) in the presence of 1% (v/v) β-mercaptoethanol. RNeasy Mini Spin columns were used for RNA isolation according to manufacturer's instructions. RNA preparations were treated twice with DNase I (Promega) in order to eliminate possible chromosomal DNA contamination. Total RNA concentration was determined with a NanoDrop ND-1000 spectrophotometer (Thermo Scientific), and quality and integrity were checked in a bioanalyzer 2100 apparatus (Agilent Technologies). Total genomic DNA (gDNA) was isolated from stationary phase cultures following the Kirby mix procedure [36].

### Microarray hybridizations

*S .coelicolor* microarrays (SCo29 print run) were obtained from the Functional Genomics Laboratory of the University of Surrey (UK). These arrays contained duplicated oligonucleotide probes for 7728 chromosomal genes (out of 7825). For each microarray hybridization, 10 pmol of Cy3-labelled cDNA obtained from total RNA were mixed with 80 pmol of Cy5-labelled genomic DNA as the common reference. Labelling, hybridization, washing and scanning conditions were carried out as indicated previously [16], except that hybridizations were extended to 72 h to improve the quality of the results [45]. Five biological replicates from independent cultures were made for each condition.

### Identification of differentially transcribed genes

Microarray data were normalized and analysed with the Bioconductor package limma [46], [47]. Spot quality weights were estimated as indicated in the section (Tables S4 and S5). As recommend by Wu et al. [48], both local and global normalizations were used. Firstly, weighted medians of log_2_ Cy3/Cy5 intensities were calculated for print-tip correction and afterwards global Loess was applied [46]. The normalized log_2_ of Cy3/Cy5 intensities is referred in this work as the M*_g_* value, which is proportional to the abundance of transcripts for a particular gene [49]. The information from within-array spot duplicates [50] and empirical array weights [51] were taken into account in the linear models [47]. Moderated t-statistics produced *p*-values which were corrected for multiple testing by the false-discovery rate method (FDR). Rank Products, a non-parametric method [52], provided another probability value, *pfp* (proportion of false positives), corrected for multiple tests. For each contrast a result was considered as statistically significant if the FDR-corrected *p*-value<0.05 or if both the *pfp* value and the uncorrected *p*-value<0.05.

### Gene expression analysis by Reverse Transcriptase PCR

Transcription was studied by using the SuperScript™ One-Step RT-PCR system with Platinum® Taq DNA polymerase (Invitrogen), using 100 ng of total RNA as template. Conditions were as follows: first strand cDNA synthesis, 45°C for 40 min followed by 94°C for 2 min; amplification, 26 or 37 cycles of [98°C for 15 sec, 60–70°C (depending of the set of primers used) for 30 sec, and 72°C for 1 min]. Primers (18–25 mers, Table S1) were designed to generate PCR products of approximately 400–600 bp. Negative controls were carried out with each set of primers and Platinum® Taq DNA polymerase in order to confirm the absence of contaminating DNA in the RNA preparations. The identity of each amplified product was corroborated by direct sequencing using one of the primers.

### Quantitative real-time PCR

Reverse transcription of total RNA was performed on selected samples with 1 µg of RNA and 12.5 ng/µl of random hexamer primer (Invitrogen) using SuperScript™ III reverse transcriptase (Invitrogen). SYBR® Premix Ex Taq™ (TaKaRa) was used for real-time quantitative PCRs, and reactions were run on a StepOnePlus Real Time PCR system (Applied Biosystems). Reactions were carried out on two biological replicates by triplicate and appropriate controls were included to verify the absence of gDNA contamination in RNA, and primer-dimer formation. Primers (see Table S6) were designed to generate PCR products between 90 and 200 bp, near the 5′ end of mRNA. The PCR reactions were initiated by incubating the sample at 95°C for 10 min followed by 40 cycles at 95°C for 15 s, 60–71°C (depending of the set of primers used) for 60 s. To check the specificity of real-time PCR reactions, a DNA melting curve analysis was performed by holding the sample at 60°C for 60 s followed by slow ramping of the temperature to 95°C. SYBR fluorescence was normalized by ROX fluorescence. Baseline and threshold values were determined by the StepOnePlus software. C_t_ values were normalized with respect to *SCO5820* (*hrdB*), the major vegetative sigma factor of *S.coelicolor*. Relative changes in gene expression were quantified using 2^−ΔΔCt^ method [53].

### Preparation of crude protein extracts and gel shift experiments

Crude protein extracts were performed as described [34]. DNA binding tests were carried out by electrophoretic mobility shift assay (EMSA) as described [54] using *phoR* [26] and *phoP* promoters as probes. A 383 bp probe containing *phoP* promoter was PCR amplified using primers 5′-GCACGGCGGGGAGGTCAC-3′ and 5′-GGCAGGCCGGGCAGCATCAG-3′. A 384 bp probe containing the upstream region of the polyketide synthase *pimS4* [54] was used as non-specific competitor.

### Microarray data availability

Microarray data generated in this study has been made available at EBI - ArrayExpress repository. Accession number: E-MEXP-2132. All microarray data is MIAME compliant.

## Supporting Information

Figure S1 **Validation of microarray results using qRT-PCR.** Correlation between qRT-PCR and microarray results for 11 different genes (see text). Samples for *S. coelicolor* Δ0877 are shown by triangles while those for *S. coelicolor* Δ7173 are indicated by squares. A least square straight line fit is also shown.
(TIF)

Table S1 **Sequence of primers employed for RT-PCR.**
(DOC)

Table S2 **Differentially expressed genes showing increased transcript levels in the LAL mutants when compared to the parental strain.** *Mc* and *p-*values for the contrasts between the indicated conditions (A: *S. coelicolor* A3(2) M145. B: *S. coelicolor* Δ0877. and C: *S. coelicolor* Δ7173 strains). Among the 322 genes with statistically significant results the table includes only those genes with a defined function that match the selected categories. Some genes are included in more than one functional category because of they are implicated in several processes. A few genes that did not meet criteria are also included (see footnote ^a^). Genes are ordered firstly by functional class, and then by chromosomal position with the aim of highlighting the coincidence of profiles among clustered genes. The primary annotation source is the StrepDB server (http://strepdb.streptomyces.org.uk). For simplicity, designations “putative” have been removed. The *p-*values BvA and CvA are indicated in bold type when found statistically significant (see Materials and Methods). When both *p*-values are statistically significant cells are shaded.
(DOC)

Table S3 **Differentially expressed genes showing decreased transcript levels in the LAL mutants when compared to the parental strain.** *Mc* and *p-*values for the contrasts between the indicated conditions (A: *S. coelicolor* A3(2) M145. B: *S. coelicolor* Δ0877. and C: *S. coelicolor* Δ7173 strains). The *p-*values BvA and CvA are indicated in bold type when found statistically significant. When both *p*-values are statistically significant cells are shaded.
(DOC)

Table S4 **Determination of the quality flag for array spots.** The Feature Extraction software quantifies spot fluorescence and provides a set of Boolean values to assess the quality of the results. These quality indicators evaluate both red and green channel data of each spot (indicators are listed in the first row). Based on previous observations of the significance of each quality indicator, we classified the indicated combinations of Boolean values into a unique quality flag. Although spots with the best quality results were flagged as “1.00”, other flag values were arbitrary.
(DOC)

Table S5 **Assigned weights to each spot flags.** To obtain spot quality weights that could be entered into the data analysis, we followed the idea behind the array weight estimation of Ritchie *et al*. [51]. In our case, the data of the 15 array hybridizations (3 conditions×5 biological replicates) were normalized, the array weights calculated, and a linear model was fitted as indicated in Materials and Methods. In this initial stage, all spots were equally weighted irrespective of their quality flags. Spots with more reproducible transcription values —less variable— between replicates indicate a higher data quality. Hence, means of spot variances for each flag group were calculated. Spot weights were simply obtained by the normalized inverse of the mean variance.
(DOC)

Table S6 **Sequence of primers used for qPCR.**
(DOC)

Appendix S1 **Complete list of** ***Mc*** **and** ***p-*** **values for all genes passing the statistical tests are provided in an Excel spreadsheet.**
(XLS)
