# Supplementary figures and images for: LAL Regulators SCO0877 and SCO7173 as Pleiotropic Modulators of Phosphate Starvation Response and Actinorhodin Biosynthesis in Streptomyces coelicolor
Source: PLoS One. 2012 Feb 20;7(2):e31475. doi: 10.1371/journal.pone.0031475 (PMC3282765; doi:10.1371/journal.pone.0031475)

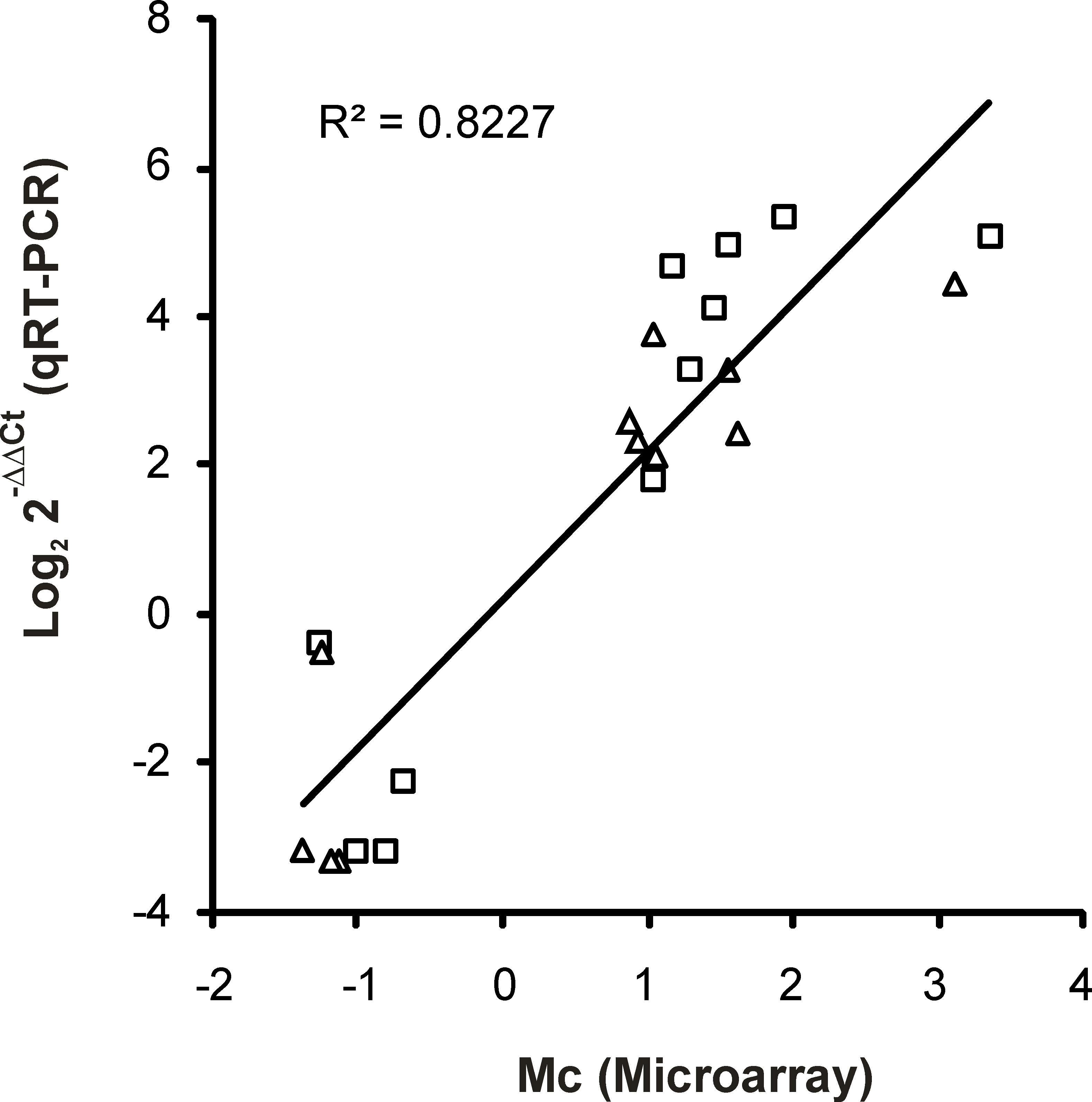

Supplement: Figure S1 — Validation of microarray results using qRT-PCR. Correlation between qRT-PCR and microarray results for 11 different genes (see text). Samples for S. coelicolor Δ0877 are shown by triangles while those for S. coelicolor Δ7173 are indicated by squares. A least square straight line fit is also shown. (TIF) [file pone.0031475.s001.tif]
