# Supplementary material for: LAL Regulators SCO0877 and SCO7173 as Pleiotropic Modulators of Phosphate Starvation Response and Actinorhodin Biosynthesis in Streptomyces coelicolor
Source: PLoS One. 2012 Feb 20;7(2):e31475. doi: 10.1371/journal.pone.0031475 (PMC3282765; doi:10.1371/journal.pone.0031475)
Supplement: Table S1 — Sequence of primers employed for RT-PCR. (DOC) [file pone.0031475.s002.doc]

**Table S1:** Primers for RT-PCR.

| **Primer** | **Sequence 5´-3´** | **Description** |
| --- | --- | --- |
| 0132-S | CCCCCTGTTCTGCCTGGTGGAC | Forward primer for SCO0132 |
| 0132-AS | AGCTGCCGTGCCGCCCGCCTGAG | Reverse primer for SCO0132 |
| 0877-S | GGGCGACCGCTTCCTTCTG | Forward primer for SCO0877 |
| 0877-AS | AGCCCACTCCCCTCCAACTCCG | Reverse primer for SCO0877 |
| 1331-S | CGGGAGCGCGGAACGAGAC | Forward primer for SCO1331 |
| 1331-AS | CGCCCGGGTGACGAACAGAG | Reverse primer for SCO1331 |
| 1351-S | CGGCGGGCCTGGGCAAGAGT | Forward primer for SCO1351 |
| 1351-AS | CGCAGCACGCCGAGCAGGTC | Reverse primer for SCO1351 |
| 5065-S | CAGATGCTCCGCCCAGTGCT | Forward primer for SCO5065 |
| 5065-AS | GCGCGAAGATCCCCTCCAG | Reverse primer for SCO5065 |
| 5506-S | GCCTCCCTCCCCGCCGTCGTTCC | Forward primer for SCO5506 |
| 5506-AS | GCCGGGCCATGCTCTCCTCGTCGTG | Reverse primer for SCO5506 |
| 6193-S | CCACCGAGTCCGAGTCCCATCT | Forward primer for SCO6193 |
| 6193-AS | CGAGCAGCGCCGACACCT | Reverse primer for SCO6193 |
| 6334-S | ACGGCACGGGGCGGTCAC | Forward primer for SCO6334 |
| 6334-AS | GCGATCTCAGGGTTGGTCAT | Reverse primer for SCO6334 |
| 7093-S | GCCGGTGCACTGGTCGTCTC | Forward primer for SCO7093 |
| 7093-AS | GCCAGGCGCAGCAGTTCACA | Reverse primer for SCO7093 |
| 7134-S | CAAGACCGAGCTGCTGAACCACCTG | Forward primer for SCO7134 |
| 7134-AS | CGAATCCCCCGGCCATCTCCA | Reverse primer for SCO7134 |
| 7137-A | AATACGACGTGCTGGACGGG | Forward primer for SCO7137 |
| 7137-AS | CGGTTCGGCGAGCATTCTTC | Reverse primer for SCO7137 |
| 7143-S | GCAGGGCATCGAGGCGGACAT | Forward primer for SCO7143 |
| 7143-AS | CCAGCAGAGCGAGCGGATTG | Reverse primer for SCO7143 |
| 7173-S | TGCTGCTCGTCGATGATCTCCAATG | Forward primer for SCO7173 |
| 7173-AS | CTGCAGGGCGTACACGGTCACTTCC | Reverse primer for SCO7173 |
| 7295-S | CGGCCGCGAGGACGAACT | Forward primer for SCO7295 |
| 7295-AS | CCCCCGGCCGAGCAGGAAC | Reverse primer for SCO7295 |
| hrdB-F | ACGCCCCGGCCCAGCAGTC | Forward primer for SCO5020 |
| hrdB-R | CAGGTGGCGTACGTGGAGAACTTGT | Reverse primer for SCO5020 |
