# Supplementary material for: LAL Regulators SCO0877 and SCO7173 as Pleiotropic Modulators of Phosphate Starvation Response and Actinorhodin Biosynthesis in Streptomyces coelicolor
Source: PLoS One. 2012 Feb 20;7(2):e31475. doi: 10.1371/journal.pone.0031475 (PMC3282765; doi:10.1371/journal.pone.0031475)
Supplement: Table S2 — Differentially expressed genes showing increased transcript levels in the LAL mutants when compared to the parental strain. Mc and p-values for the contrasts between the indicated conditions (A: S. coelicolor A3(2) M145. B: S. coelicolor Δ0877. and C: S. coelicolor Δ7173 strains). Among the 322 genes with statistically significant results the table includes only those genes with a defined function that match the selected categories. Some genes are included in more than one functional category because of they are implicated in several processes. A few genes that did not meet criteria are also included (see footnote a). Genes are ordered firstly by functional class, and then by chromosomal position with the aim of highlighting the coincidence of profiles among clustered genes. The primary annotation source is the StrepDB server (http://strepdb.streptomyces.org.uk). For simplicity, designations “putative” have been removed. The p-values BvA and CvA are indicated in bold type when found statistically significant (see Materials and Methods). When both p-values are statistically significant cells are shaded. (DOC) [file pone.0031475.s003.doc]

Table S2. Differentially expressed genes showing increased transcript levels in the LAL mutants when compared to the parental strain.

| **Category** | **Systematic name** | **Gene name** | **Product** | ***Mc* BvA** | ***Mc* CvA** | ***p-*value BvA** | | ***p-*value CvA** |
| --- | --- | --- | --- | --- | --- | --- | --- | --- |
| Amino acids metabolism. Transcription and translation | SCO0613 | *arcA* | arginine deiminase | **0.42** | **0.76** | 0.0359 | | **0.0004** |
|  | SCO0992 |  | cysteine synthase | **0.79** | **0.61** | **0.0004** | | 0.0048 |
|  | SCO1054 |  | aminotransferase | **0.78** | **0.67** | **0.0002** | | **0.0012** |
|  | SCO1222 |  | amidinotransferase | **1.31** | **0.63** | **0.0000** | | 0.0030 |
|  | SCO1276 | *sigJ* | RNA polymerase ECF sigma factor | **1.16** | **0.78** | **0.0003** | | 0.0115 |
|  | SCO1657 |  | methionine synthase | **0.84** | **0.73** | **0.0000** | | **0.0003** |
|  | SCO1916 | *dapD* | transferase | **1.07** | **1.25** | 0.0018 | | **0.0005** |
|  | SCO2018 | *pepN* | aminopeptidase N | **0.75** | **1.07** | 0.0056 | | **0.0002** |
|  | SCO3397 |  | integral membrane lysyl-tRNA synthetase | **0.98** | **1.29** | **0.0002** | | **0.0000** |
|  | SCO3801 |  | aspartyl aminopeptidase | **0.66** | **1.08** | 0.0047 | | **0.0000** |
|  | SCO4366 |  | phosphoserine aminotransferase | **0.64** | **0.43** | **0.0005** | | 0.0157 |
|  | SCO4384 |  | enoyl CoA hydratase | **0.32** | **1.44** | 0.2886 | | **0.0000** |
| Nucleotide and coenzyme metabolism. DNA replication, recombination and repair | SCO1167 |  | helicase | **1.39** | **1.01** | **0.0000** | | **0.0000** |
|  | SCO1202 |  | DNA ligase | **1.01** | **0.70** | **0.0003** | | 0.0100 |
|  | SCO2162 |  | quinolinate synthetase (fragment) | **1.13** | **0.67** | **0.0000** | | 0.0061 |
|  | SCO3383 | *panC* | pantoate-amino acid ligase | **0.91** | **0.67** | **0.0004** | | 0.0068 |
|  | SCO3401 | *folK* | hydroxymethyldihydropteridina pyrophosphokinase | **0.48** | **0.90** | 0.0209 | | **0.0001** |
|  | SCO4079 | *purL* | phosphoribosyl formylglycinamidine synthase II | **0.63** | **0.56** | **0.0008** | | 0.0028 |
|  | SCO4971 |  | xanthine dehydrogenase | **0.86** | **1.32** | 0.0073 | | **0.0001** |
|  | SCO5465 |  | NADPH oxidoreductase coenzyme F420 dependent | **0.67** | **-0.01** | **0.0007** | | 0.9370 |
| Respiration and energy production | SCO1419 | *ugpQ2* | glicerophosphoryl diester phosphodiesterase | **1.70** | **0.97** | | **0.0000** | 0.0031 |
|  | SCO1955 |  | Rieske iron sulphur binding protein | **0.70** | **1.18** | | 0.0385 | **0.0011** |
|  | SCO1968 | *glpQ2* | secreted glycerophosphoryl diester phosphodiesterase | **3.35** | **3.13** | | **0.0000** | **0.0000** |
|  | SCO4948 | *narH3* | nitrate reductase beta chain NarH3 | **0.67** | **0.99** | | 0.0017 | **0.0000** |
|  | SCO5424 | *ackA* | acetate kinase | **0.82** | **0.52** | | **0.0003** | 0.0156 |
|  | SCO5842 |  | acyl-CoA synthetase | **0.72** | **1.07** | | 0.0089 | **0.0003** |
| Cell envelope biosynthesis and morphological differentiation | SCO1950 | *whiA* | sporulation regulatory protein | **1.78** | **1.02** | | **0.0030** | 0.0865 |
|  | SCO2187 |  | acetylmuramoyl-L-alanine amidase | **0.96** | **0.86** | | **0.0000** | **0.0002** |
|  | SCO2328 |  | D-ala-D-ala dipeptidase | **0.83** | **1.40** | | 0.0867 | **0.0066** |
|  | SCO2924 | *ssgG* | sporulation regulator | **1.23** | **0.37** | | **0.0000** | 0.1680 |
|  | SCO3846 | *ftsW* | FtsW/RodA/SpoVE-family cell cycle protein | **1.31** | **1.58** | | **0.0000** | **0.0000** |
|  | SCO4880 | *neuA* | transferase | **0.78** | **0.34** | | **0.0005** | 0.1048 |
|  | SCO4881 | *neuB^a)^* | polisaccharide biosynthesis related protein | **0.93** | **0.33** | | 0.0020 | 0.2512 |
|  | SCO5039 |  | penicillin-binding protein pbp2 | **0.40** | **0.75** | | 0.0349 | **0.0003** |
|  | SCO5582 | *nsdA* | negative regulator of differentiation | **0.93** | **0.32** | | **0.0003** | 0.1732 |
|  | SCO6060 | *murC* | UDP-N-acetylmuramoyl-L-alanine ligase | **0.61** | **0.93** | | 0.0015 | **0.0000** |
|  | SCO6682 | *ramS* | developmental protein SC5A7.32 | **0.60** | **1.01** | | 0.0292 | **0.0007** |
|  | SCO6691 |  | phospholipase C | **0.91** | **0.29** | | **0.0003** | 0.2043 |
|  | SCO7306 | *wblK* | WhiB-family transcriptional regulator | **0.48** | **1.23** | | 0.0611 | **0.0000** |
| Carbohydrate metabolism | SCO4209 | *pgm1* | phophoglycerate mutase | **0.82** | **1.03** | | 0.0019 | **0.0002** |
|  | SCO6232 |  | beta-mannosidase | **0.11** | **0.79** | | 0.5461 | **0.0002** |
|  | SCO6234 | *manA* | secreted beta-mannosidase | **0.33** | **0.66** | | 0.0528 | **0.0003** |
|  | SCO6548 |  | secreted cellulase | **0.20** | **0.79** | | 0.2939 | **0.0003** |
| Lipid metabolism | SCO0330 |  | 3-ketoacyl-ACP/CoA reductase | **0.91** | **0.70** | | **0.0001** | 0.0021 |
|  | SCO0920 |  | acylglycerol-3-phosphate O-acyltransferase | **1.77** | **1.00** | | **0.0000** | **0.0000** |
|  | SCO1048 |  | phospholipase A2 | **1.47** | **1.32** | | **0.0001** | **0.0003** |
|  | SCO1209 |  | short chain acyl-CoA dehydrogenase | **1.18** | **1.04** | | **0.0000** | **0.0001** |
|  | SCO2194 | *lipA* | lipoic acid synthetase | **0.82** | **0.72** | | **0.0001** | **0.0005** |
|  | SCO4234 |  | 2-C-methyl-D-erythritol 2.4-cyclodiphosphate synthase | **0.55** | **1.24** | | 0.1071 | **0.0013** |
|  | SCO4384 |  | enoyl CoA hydratase | **0.32** | **1.44** | | 0.2886 | **0.0000** |
|  | SCO6470 |  | MaoC-like protein (Acyl dehydratase) | **0.69** | **1.15** | | 0.0016 | **0.0000** |
| Phosphate starvation response | SCO0920 |  | acylglycerol-3-phosphate O-acyltransferase | **1.77** | **1.00** | | **0.0000** | **0.0000** |
|  | SCO1048 |  | phospholipase A2 | **1.47** | **1.32** | | **0.0001** | **0.0003** |
|  | SCO1196*^b)^* |  | Tat dependent secreted protein | **1.94** | **1.62** | | **0.0000** | **0.0000** |
|  | SCO1419 | *ugpQ2* | glycerophosphoryl diester phosphodiesterase | **1.70** | **0.97** | | **0.0000** | 0.0031 |
|  | SCO1633 | *tatA* | Tat dependent secreted protein | **1.00** | **1.22** | | **0.0002** | **0.0000** |
|  | SCO1845 | *pitH2^a,c)^* | low-affinity phosphate transport protein | **0.91** | **0.86** | | 0.0252 | 0.0401 |
|  | SCO1968 | *glpQ2^d)^* | secreted glycerophosphoryl diester phosphodiesterase | **3.35** | **3.13** | | **0.0000** | **0.0000** |
|  | SCO2198 | *glnA^a,e)^* | glutamine synthetase I | **0.61** | **0.25** | | 0.0045 | 0.2266 |
|  | SCO2286 | *phoA^a,f)^* | alkaline phosphatase | **0.86** | **0.32** | | 0.0056 | 0.2752 |
|  | SCO2428 |  | phosphate binding protein (secreted protein) | **1.57** | **1.31** | | **0.0000** | **0.0001** |
|  | SCO4140 | *pstA^d)^* | phosphate ABC transport system permease protein | **1.03** | **0.71** | | **0.0001** | 0.0032 |
|  | SCO4141 | *pstC^a,d)^* | phosphate ABC transport system permease protein | **0.64** | **-0.07** | | 0.0143 | 0.7711 |
|  | SCO4142 | *pstS^a,d)^* | phosphate-binding protein precursor | **0.97** | **0.79** | | 0.0012 | 0.0069 |
|  | SCO4209 | *pgm1* | phosphoglycerate mutase | **0.82** | **1.03** | | 0.0019 | **0.0002** |
|  | SCO4226 |  | hypothetical protein | **0.91** | **0.54** | | **0.0002** | 0.0180 |
|  | SCO4227 | *mtpA* | metallothionein | **1.15** | **0.28** | | **0.0000** | 0.2728 |
|  | SCO4228 | *phoU^d)^* | Phosphate transport system regulatory protein | **1.45** | **0.87** | | **0.0000** | **0.0013** |
|  | SCO4229 | *phoR^d)^* | PhoR sensor kinase | **1.29** | **0.93** | | **0.0000** | **0.0007** |
|  | SCO4230 | *phoP^d)^* | PhoP response regulator | **1.17** | **1.04** | | **0.0000** | **0.0001** |
|  | SCO4880 | *neuA^b)^* | transferase | **0.78** | **0.34** | | **0.0005** | 0.1048 |
|  | SCO4881 | *neuB^a,b)^* | N-acylneuraminate-9-phosphate synthase | **0.93** | **0.33** | | 0.0020 | 0.2512 |
|  | SCO5424 | *ackA^g)^* | Acetate kinase | **0.82** | **0.52** | | **0.0003** | 0.0156 |
|  | SCO5746 |  | Hypothetical protein SC7C7.01 | **1.49** | **1.55** | | **0.0000** | **0.0000** |
|  | SCO6691 |  | Tat dependent secreted protein | **0.91** | **0.29** | | **0.0003** | 0.2043 |
|  | SCO7344 |  | secreted protein | **1.57** | **0.98** | | **0.0000** | 0.0025 |
|  | SCO7631 |  | Tat dependent secreted protein | **0.73** | **0.99** | | 0.0047 | **0.0003** |
|  | SCO7697 | *phy^b)^* | secreted phytase | **1.09** | **0.71** | | **0.0001** | 0.0072 |
| Regulation | SCO0148 |  | transcriptional regulatory protein | **1.17** | **0.79** | | **0.0000** | **0.0007** |
|  | SCO0275 |  | transcriptional repressor protein | **0.85** | **0.38** | | **0.0001** | 0.0636 |
|  | SCO0471 |  | AraC-family transcriptional regulator | **0.86** | **1.10** | | 0.0019 | **0.0002** |
|  | SCO0605 |  | AraC-family transcriptional regulator | **0.75** | **0.88** | | 0.0043 | **0.0012** |
|  | SCO0702 | *abaA-orfA* | pleiotropic regulator | **0.87** | **0.16** | | **0.0413** | 0.6953 |
|  | SCO1066 |  | LacI-family transcriptional regulator | **1.07** | **1.69** | | **0.0003** | **0.0000** |
|  | SCO1119 |  | AsnC-family transcriptional regulatory protein | **0.45** | **1.01** | | 0.0746 | **0.0002** |
|  | SCO1370 |  | two component system DNA binding response regulator | **0.93** | **1.02** | | 0.0020 | **0.0009** |
|  | SCO1950 | *whiA* | sporulation regulatory protein | **1.78** | **1.02** | | **0.0030** | 0.0865 |
|  | SCO2374 |  | TetR-family transcriptional regulator | **0.87** | **0.56** | | **0.0005** | 0.0195 |
|  | SCO2775 |  | TetR-family regulatory protein | **1.26** | **0.88** | | **0.0002** | 0.0076 |
|  | SCO2924 | *ssgG* | sporulation regulator | **1.23** | **0.37** | | **0.0000** | 0.1680 |
|  | SCO3653 |  | two-component system response regulator | **0.53** | **0.67** | | 0.0034 | **0.0004** |
|  | SCO3750 |  | two- component sensor histidine kinase | **0.69** | **1.13** | | 0.0261 | **0.0007** |
|  | SCO3975 |  | regulator | **1.52** | **1.56** | | **0.0005** | **0.0004** |
|  | SCO3979 |  | TetR-family transcriptional regulator | **0.59** | **0.76** | | 0.0067 | **0.0009** |
|  | SCO4122 |  | MarR-family transcriptional regulator | **0.74** | **0.36** | | **0.0007** | 0.0793 |
|  | SCO4198 |  | DNA-binding protein | **0.77** | **0.67** | | **0.0008** | 0.0032 |
|  | SCO4228 | *phoU^c)^* | phosphate transport system regulator | **1.45** | **0.87** | | **0.0000** | **0.0013** |
|  | SCO4229 | *phoR^d)^* | PhoR sensor kinase | **1.29** | **0.93** | | **0.0000** | **0.0007** |
|  | SCO4230 | *phoP^d)^* | response regulator | **1.17** | **1.04** | | **0.0000** | **0.0001** |
|  | SCO4263*^h)^* |  | LuxR-family transcriptional regulator | **0.81** | **0.82** | | **0.0004** | **0.0005** |
|  | SCO4276 | *senR* | response regulatory protein | **0.81** | **1.11** | | 0.0068 | **0.0004** |
|  | SCO4434 |  | LysR-family transcriptional regulator | **0.45** | **0.81** | | 0.0439 | **0.0008** |
|  | SCO4906 | *afsQ2* | sensor kinase protein | **0.62** | **0.83** | | 0.0046 | **0.0003** |
|  | SCO4907 | *afsQ1^a)^* | transcriptional regulatory protein | **0.70** | **0.24** | | 0.0087 | 0.3597 |
|  | SCO5532 |  | TetR-family transcriptional regulator | **0.41** | **1.27** | | 0.1847 | **0.0003** |
|  | SCO5582 | *nsdA* | sporulation regulator | **0.93** | **0.32** | | **0.0003** | 0.1732 |
|  | SCO5917 |  | MerR -family transcriptional regulator | **0.71** | **1.20** | | 0.0411 | **0.0012** |
|  | SCO7054 |  | PadR-like family transcriptional regulator | **1.05** | **1.02** | | **0.0000** | **0.0000** |
|  | SCO7306 | *wblK* | WhiB-family transcriptional regulator | **0.48** | **1.23** | | 0.0611 | **0.0000** |

a) Gene included because its transcription profile matches those of genes functionally related.

b) PhoP activates the transcription of this gene directly according to Sola-Landa et al. [Nucleic Acids Res 36: 1358-1368].

c) PhoP activates the transcription of this gene directly according to Santos-Beneit et al. [Microbiology 154: 2356-2370].

d) PhoP activates the transcription of this gene directly [16].

e) PhoP negatively regulates the transcription of the *glnA* gene by direct binding to its promoter region and also indirectly by repression of the *glnR* gene according to Rodríguez-García et al. [Nucleic Acids Res 37: 3230-3242].

f) PhoP activates the transcription of this gene directly according to Apel et al. [Microbiology 153: 3527-3537].

g) PhoB controls the transcription of this gene in *E. coli* [J Bacteriol 174: 2124-2130].

h) A PhoP binding site is placed at the promoter region of this gene [Nucleic Acids Res 36: 1358-1368].
