# Supplementary material for: LAL Regulators SCO0877 and SCO7173 as Pleiotropic Modulators of Phosphate Starvation Response and Actinorhodin Biosynthesis in Streptomyces coelicolor
Source: PLoS One. 2012 Feb 20;7(2):e31475. doi: 10.1371/journal.pone.0031475 (PMC3282765; doi:10.1371/journal.pone.0031475)
Supplement: Table S3 — Differentially expressed genes showing decreased transcript levels in the LAL mutants when compared to the parental strain. Mc and p-values for the contrasts between the indicated conditions (A: S. coelicolor A3(2) M145. B: S. coelicolor Δ0877. and C: S. coelicolor Δ7173 strains). The p-values BvA and CvA are indicated in bold type when found statistically significant. When both p-values are statistically significant cells are shaded. (DOC) [file pone.0031475.s004.doc]

Table S3. Differentially expressed genes showing decreased transcript levels in the LAL mutants when compared to the parental strain.

| **Category** | **Systematic name** | **Gene name** | **Product** | ***Mc* BvA** | ***Mc* CvA** | ***p-*value BvA** | | ***p-*value CvA** |
| --- | --- | --- | --- | --- | --- | --- | --- | --- |
| Amino acids metabolism. Transcription and translation | SCO1492 |  | peptidase | **-0.18** | **-0.92** | 0.4819 | | **0.0009** |
|  | SCO1505 | *rpsD* | 30S ribosomal protein S4 | **-0.31** | **-1.20** | 0.2140 | | **0.0000** |
|  | SCO2954 | *sigU* | RNA polymerase sigma factor | **-0.85** | **-1.27** | 0.0370 | | **0.0029** |
|  | SCO3892 | *sigT* | RNA polymerase sigma factor | **-0.61** | **-0.92** | 0.0038 | | **0.0001** |
|  | SCO4649 | *rplA* | 50S ribosomal protein L1 | **-0.06** | **-0.65** | 0.7334 | | **0.0008** |
|  | SCO4659 | *rpsL* | 30S ribosomal protein S12 | **-0.10** | **-1.17** | 0.7476 | | **0.0007** |
|  | SCO4720 | *rpmD* | 50S ribosomal protein L30 | **-0.02** | **-1.50** | 0.9529 | | **0.0006** |
|  | SCO4769 | *ECF* | ECF sigma factor | **-0.44** | **-1.03** | 0.0817 | | **0.0002** |
|  | SCO4908 | *sigQ* | RNA polymerase sigma factor | **-0.10** | **-1.25** | 0.7094 | | **0.0000** |
|  | SCO4958 | *metB* | cystathionine gamma-synthase | **-0.64** | **-1.04** | 0.0022 | | **0.0000** |
|  | SCO6743 |  | transcriptional accessory protein | **-0.27** | **-0.62** | 0.1372 | | **0.0014** |
| Nucleotide and coenzyme metabolism. DNA replication, recombination and repair | SCO1522 | *pdxT* | glutamine amidotransferase subunit pdxT | **-0.06** | **-0.88** | 0.7860 | | **0.0003** |
|  | SCO1958 | *uvrA* | ABC excision nuclease subunit A | **-0.64** | **-1.10** | 0.0416 | | **0.0011** |
|  | SCO3319 | *hemA* | glutamyl-tRNA reductase | **-0.55** | **-0.81** | 0.0065 | | **0.0002** |
|  | SCO3347 |  | AP endonuclease | **-0.32** | **-0.73** | 0.0978 | | **0.0005** |
|  | SCO3873 | *gyrA* | DNA gyrase subunit A | **-0.83** | **-1.62** | 0.0189 | | **0.0000** |
|  | SCO5166 |  | helicase | **-0.31** | **-0.95** | 0.2297 | | **0.0007** |
|  | SCO5439 |  | helicase | **-0.63** | **-1.01** | 0.0215 | | **0.0006** |
|  | SCO5770 | *recX* | recombination regulatory protein | **-0.88** | **-1.08** | **0.0004** | | **0.0000** |
| Respiration and energy production | SCO1934 | *cyoE* | cytochrome oxidase assembly factor | **-1.15** | **-0.12** | | **0.0007** | 0.7079 |
|  | SCO5368 | *atpE* | ATP synthase C chain | **-0.36** | **-1.01** | | 0.1929 | **0.0010** |
|  | SCO5372 | *atpG* | ATP synthase gamma chain | **-0.19** | **-1.08** | | 0.4572 | **0.0002** |
| Cell envelope biosynthesis and morphological differentiation | SCO0399 |  | pyruvyl transferase peptidoglycan biosynthesis | **-0.37** | **-1.06** | | 0.2179 | **0.0011** |
|  | SCO2953 | *rsuA* | anti-sigma factor | **-0.79** | **-1.19** | | 0.0261 | **0.0015** |
|  | SCO2954 | *sigU* | RNA polymerase sigma factor | **-0.85** | **-1.27** | | 0.0370 | **0.0029** |
|  | SCO6753 |  | nucleotide sugar-1-phosphate transferase | **-0.72** | **-0.83** | | 0.0009 | **0.0002** |
| Carbohydrate metabolism | SCO0766 |  | secreted beta-galactosidase | **-0.44** | **-0.93** | | 0.0347 | **0.0001** |
|  | SCO1935 | *tktA1* | transketolase A | **0.00** | **-0.90** | | 0.9945 | **0.0003** |
|  | SCO6374 |  | membrane sugar transferase | **-0.58** | **-0.92** | | 0.0115 | **0.0002** |
| Secondary metabolites | SCO5073 | *actVI-orf2* | enoyl reductase | **-1.01** | **-1.14** | | **0.0002** | **0.0001** |
|  | SCO5075 | *actVI-orf4* | oxidoreductase | **-0.80** | **-1.19** | | 0.0012 | **0.0000** |
|  | SCO5080 | *actVA5* | hydrolase | **-0.79** | **-1.06** | | 0.0107 | **0.0011** |
|  | SCO5083 | *actII-2* | actinorhodin transporter | **-0.68** | **-1.39** | | 0.0456 | **0.0002** |
|  | SCO5088 | *actI-orf2* | actinorhodin polyketide β-ketoacyl synthase β subunit | **-0.86** | **-1.16** | | 0.0123 | **0.0014** |
|  | SCO6277 | *cpkE* | epoxidase hydrolase | **-0.29** | **-0.77** | | 0.1689 | **0.0007** |
| Regulation | SCO2753 |  | LacI-family transcriptional regulator | **-0.34** | **-0.77** | | 0.0920 | **0.0005** |
|  | SCO2953 | *rsuA* | anti-sigma factor | **-0.79** | **-1.19** | | 0.0261 | **0.0015** |
|  | SCO2954 | *sigU* | RNA polymerase sigma factor | **-0.85** | **-1.27** | | 0.0370 | **0.0029** |
|  | SCO3857 |  | regulatory protein | **-0.74** | **-1.02** | | 0.0081 | **0.0006** |
|  | SCO4425 | *afsS^a,b)^* | sigma-like protein | **-0.55** | **-1.17** | | 0.2337 | 0.0083 |
|  | SCO4908 | *sigQ* | RNA polymerase sigma factor | **-0.10** | **-1.25** | | 0.7094 | **0.0000** |
|  | SCO4920 |  | DeoR-family transcriptional regulator | **-0.22** | **-1.48** | | 0.5297 | **0.0002** |
|  | SCO5770 | *recX* | recombination regulatory protein | **-0.88** | **-1.08** | | **0.0004** | **0.0000** |
|  | SCO7089 |  | two-component system sensor kinasa | **-0.82** | **-0.28** | | **0.0008** | 0.2299 |

a) Gene included because its transcription profile matches those of genes functionally related.

b) PhoP negatively controls the transcription of this gene by direct binding [29].
