# Supplementary material for: LAL Regulators SCO0877 and SCO7173 as Pleiotropic Modulators of Phosphate Starvation Response and Actinorhodin Biosynthesis in Streptomyces coelicolor
Source: PLoS One. 2012 Feb 20;7(2):e31475. doi: 10.1371/journal.pone.0031475 (PMC3282765; doi:10.1371/journal.pone.0031475)
Supplement: Table S4 — Determination of the quality flag for array spots. The Feature Extraction software quantifies spot fluorescence and provides a set of Boolean values to assess the quality of the results. These quality indicators evaluate both red and green channel data of each spot (indicators are listed in the first row). Based on previous observations of the significance of each quality indicator, we classified the indicated combinations of Boolean values into a unique quality flag. Although spots with the best quality results were flagged as “1.00”, other flag values were arbitrary. (DOC) [file pone.0031475.s005.doc]

**Table S4.** Determination of the quality flag for array spots

| **glsPosAndSignif** | **rlsPosAndSignif** | **glsWellAboveBG** | **rlsWellAboveBG** | **glsFeatNonUnifOL** | **rlsFeat NonUnifOL** | **glsBGNonUnifOL** | **rlsBGNonUnifOL** | **glsSaturated** | **RlsSaturated** | **Flag** |
| --- | --- | --- | --- | --- | --- | --- | --- | --- | --- | --- |
| 1 | 1 | 1 | 1 | 0 | 0 | 0 | 0 | 0 | 0 | 1.00 |
|  | 0 |  |  |  |  |  |  |  |  | 0.01 |
|  |  |  | 0 | 0 | 0 | 1 |  |  |  | 1.00 |
|  |  |  | 0 | 0 | 1 | 0 |  |  |  |  |
|  |  |  | 0 | 1 | 0 | 0 |  |  |  |  |
|  |  |  | 1 | 0 | 0 | 0 |  |  |  |  |
|  |  |  | 0 | 1 | 1 | 0 |  |  |  |  |
|  |  |  | 1 | 0 | 0 | 1 |  |  |  |  |
|  |  |  | 0 | 0 | 1 | 1 |  |  |  |  |
|  |  |  | 0 | 0 | 0 | 0 |  |  |  | 0.95 |
|  |  |  | 1 | 1 | 0 | 0 |  |  |  | 0.80 |
|  |  |  | 1 | 0 | 1 | 0 |  |  |  | 0.70 |
|  |  |  | 0 | 1 | 0 | 1 |  |  |  | 0.60 |
|  |  |  | 0 | 1 | 1 | 1 |  |  |  | 0.40 |
|  |  |  | 1 | 0 | 1 | 1 |  |  |  |  |
|  |  |  | 1 | 1 | 0 | 1 |  |  |  |  |
|  |  |  | 1 | 1 | 1 | 0 |  |  |  |  |
|  |  |  | 1 | 1 | 1 | 1 |  |  |  | 0.30 |

The Feature Extraction software quantifies spot fluorescence and provides a set of Boolean values to assess the quality of the results. These quality indicators evaluate both red and green channel data of each spot (indicators are listed in the first row). Based on previous observations of the significance of each quality indicator, we classified the indicated combinations of Boolean values into a unique quality flag. Although spots with the best quality results were flagged as "1.00", other flag values were arbitrary.
