# Supplementary material for: LAL Regulators SCO0877 and SCO7173 as Pleiotropic Modulators of Phosphate Starvation Response and Actinorhodin Biosynthesis in Streptomyces coelicolor
Source: PLoS One. 2012 Feb 20;7(2):e31475. doi: 10.1371/journal.pone.0031475 (PMC3282765; doi:10.1371/journal.pone.0031475)
Supplement: Table S5 — Assigned weights to each spot flags. To obtain spot quality weights that could be entered into the data analysis, we followed the idea behind the array weight estimation of Ritchie et al. [51]. In our case, the data of the 15 array hybridizations (3 conditions×5 biological replicates) were normalized, the array weights calculated, and a linear model was fitted as indicated in Materials and Methods. In this initial stage, all spots were equally weighted irrespective of their quality flags. Spots with more reproducible transcription values —less variable— between replicates indicate a higher data quality. Hence, means of spot variances for each flag group were calculated. Spot weights were simply obtained by the normalized inverse of the mean variance. (DOC) [file pone.0031475.s006.doc]

**Table S5.** Assigned weights to each spot flags

| **Quality flag** | **Mean of variances** | **Quality weight** | **Percentage of total spots** |
| --- | --- | --- | --- |
| 1.00 | 5.72 | 1.000 | 92.4 |
| 0.95 | 11.95 | 0.479 | 1.30 |
| 0.80 | 6.50 | 0.880 | 4.70 |
| 0.70 | 7.36 | 0.778 | 0.004 |
| 0.60 | 5.99 | 0.956 | 0.49 |
| 0.40 | 5.87 | 0.975 | 0.68 |
| 0.30 | 7.24 | 0.791 | 0.13 |
| 0.01 | 7.12 | 0.804 | 0.26 |

To obtain spot quality weights that could be entered into the data analysis, we followed the idea behind the array weight estimation of Ritchie *et al*. [51]. In our case, the data of the 15 array hybridizations (3 conditions x 5 biological replicates) were normalized, the array weights calculated, and a linear model was fitted as indicated in Materials and Methods. In this initial stage, all spots were equally weighted irrespective of their quality flags. Spots with more reproducible transcription values —less variable— between replicates indicate a higher data quality. Hence, means of spot variances for each flag group were calculated. Spot weights were simply obtained by the normalized inverse of the mean variance.
