# Supplementary material for: LAL Regulators SCO0877 and SCO7173 as Pleiotropic Modulators of Phosphate Starvation Response and Actinorhodin Biosynthesis in Streptomyces coelicolor
Source: PLoS One. 2012 Feb 20;7(2):e31475. doi: 10.1371/journal.pone.0031475 (PMC3282765; doi:10.1371/journal.pone.0031475)
Supplement: Table S6 — Sequence of primers used for qPCR. (DOC) [file pone.0031475.s007.doc]

**Table S6:** Primers for qPCR.

| **Primer** | **Sequence 5´-3´** | **Description** |
| --- | --- | --- |
| Q-hrdBD | CGCGGCATGCTCTTCCT | Forward primer for SCO5820 |
| Q-hrdBR | AGGTGGCGTACGTGGAGAAC | Reverse primer for SCO5820 |
| Q-1196D | ACCCTGGAGTCGCTCTACCT | Forward primer for SCO1196 |
| Q-1196R | CTCACCCTCGGTCTTGATGT | Reverse primer for SCO1196 |
| Q-1209D | AGCTGAGCGACGAACAGAAG | Forward primer for SCO1209 |
| Q-1209R | AGGGAGTAGATGCCGACCTT | Reverse primer for SCO1209 |
| Q-1968D | GTACGCGCCGGAGAACAC | Forward primer for SCO1968 |
| Q-1968R | GAGAGCGGTCGGGGAAGA | Reverse primer for SCO1968 |
| Q-4228D | CCTGCACGCGACCATCCT | Forward primer for SCO4228 |
| Q-4228R | TCGCGTCGTCGTCCTGCT | Reverse primer for SCO4228 |
| Q-4229D | GACACCGTCCTCTCCGTACT | Forward primer for SCO4229 |
| Q-4229R | CTCGACCTGCCGTATCTCTC | Reverse primer for SCO4229 |
| Q-4230D | GCCGACCTCGTGCTCCTC | Forward primer for SCO4230 |
| Q-4230R | AGTCGTCGGCCCCTATCTCC | Reverse primer for SCO4230 |
| Q-5073D | CGGCCGGCTGGTGTTCTA | Forward primer for SCO5073 |
| Q-5073R | AGCCGGTGACGTACTTGATGC | Reverse primer for SCO5073 |
| Q-5075D | CCGGCGAGCACAACCAC | Forward primer for SCO5075 |
| Q-5075R | GACACGGCGAGGGCTTCC | Reverse primer for SCO5075 |
| Q-5083D | CGGCTGGGCGACATCTAC | Forward primer for SCO5083 |
| Q-5083R | CTTGGGCGGGAACATTTG | Reverse primer for SCO5083 |
| Q-5746D | CTTCGGGAACGTGGAAGTC | Forward primer for SCO5746 |
| Q-5746R | GGTGCAGAGAGCCCATGT | Reverse primer for SCO5746 |
| Q-7318D | CGGCGAGATCCTGGTCAAG | Forward primer for SCO7318 |
| Q-7318R | AGCGGCGCCGTGTAGAAC | Reverse primer for SCO7318 |
